# Prevalence of dental caries in children and adolescents with type 1 diabetes: a systematic review and meta-analysis

**DOI:** 10.1186/s12903-019-0903-5

**Published:** 2019-09-14

**Authors:** Yan Wang, Lin Xing, Hui Yu, LiJuan Zhao

**Affiliations:** Yan Tai Stomatological Hospital, No. 142 Beida Street Zhifu District, Yantai, 264008 Shandong China

**Keywords:** Adolescent, Caries, Children, Diabetes, Meta-analysis, Prevalence

## Abstract

**Background:**

Dental caries and type 1 diabetes are responsible for a large burden of global disease; however, the exact prevalence of dental caries among children and adolescents with type 1 diabetes remains controversial, and no quantitative meta-analysis exists. Thus, we performed a meta-analysis to evaluate the prevalence of dental caries among children and adolescents with type 1 diabetes.

**Methods:**

We performed a systematic search strategy using PubMed, EMBASE and China National Knowledge Infrastructure for relevant studies investigating the prevalence of dental caries in children and adolescents with type 1 diabetes from July 1971 until December 2018. The pooled prevalence with 95% confidence intervals (95%CIs) and subgroup analyses were calculated using a random effects model.

**Results:**

After screening 358 non-duplicated articles, a total of 10 articles involving 538 individuals were included. The overall prevalence of dental caries among children and adolescents with type 1 diabetes was 67% (95% CI: 0.56–0.77%; *I*^2^ = 83%). The prevalence was highest in South America (84%) and lowest in diabetic patients with good metabolic control (47%).

**Conclusions:**

The prevalence of dental caries was high among children and adolescents with type 1 diabetes. Screening and preventive treatment should be included in dental clinical routines for diabetic children and adolescents, especially in those with poor metabolic control.

**Electronic supplementary material:**

The online version of this article (10.1186/s12903-019-0903-5) contains supplementary material, which is available to authorized users.

## Introduction

Type 1 diabetes mellitus is a chronic autoimmune disease characterized by the destruction of pancreatic beta cells and insulin deficiency, and affects over half a million children worldwide [1]. The prevalence and incidence of type 1 diabetes is increasing, especially in European countries [2]. Numerous epidemiological studies have reported that type 1 diabetes increases the risk for cardiovascular diseases [3], kidney disease [4] and cognitive decline [5] in children and adolescents. Additionally, a growing number of studies indicate an underlying link between type 1 diabetes and oral complications, including periodontal diseases [6] and dental caries [7]. Dental caries is the most common chronic infectious disease, and has posed an international public health challenge, especially in young children [8]. Additionally, it has become a major concern as it can begin early in life, progress rapidly in those individuals who are at high risk, and often goes untreated [9]. Its consequences can lead to poor food intake, poor school performance, and mental health problems, which can affect the quality of life of the child’s family, and impact significant social and economic burdens as well [10].

Clinical caries are diagnosed by the DMFT index (D = dentine caries lesion; M = missing due to caries; F = filled; T = tooth), according to World Health Organization (WHO) criteria [11]. Although dental caries have been declining, a national survey in the United States between 2001 and 2012 showed that approximately 37% of children aged 2–8 years and 60% of adolescents aged 12–19 years had experienced dental caries in their primary teeth [12]. One goal of the WHO is to reduce the DMFT index in 2020, and in particular, the D component, in high-risk groups [13].

Screening and preventive treatment are necessary to avoid dental caries before they become incurable in the high-risk population. However, the exact prevalence of dental caries remains controversial in children and adolescents with type 1 diabetes [14–17], especially in those with poor metabolic control [18]. Previous studies reported that the prevalence of dental caries in children and adolescents varies between 36% in Iran [19] and 92% in Chile [20]. Moreover, several studies have showed that a higher prevalence was observed in diabetic adolescents with poor metabolic control compared to patients with good metabolic control [18, 21]. Therefore, we performed a meta-analysis using data available from access reports to estimate the overall prevalence of dental caries in children and adolescents with type 1 diabetes.

## Methods

### Search strategy

This meta-analysis was conducted and reported according to the Preferred Reporting Items for Systematic Reviews and Meta-Analysis statement (Additional file 2) [22]. Major databases including PubMed, EMBASE and China National Knowledge Infrastructure were searched from inception to 28 December 2018 for terms that contained the keywords “dental caries” and “type 1 diabetes” in multiple combinations. The search strategy was conducted without language restrictions (Additional file 1). We also manually reviewed the reference lists of all studies of interest to identify additional studies.

### Study eligibility

The inclusion criteria were as follows: 1) cross-sectional studies or the first evaluation of longitudinal studies; 2) studies that were conducted among children (under 12 years old) and adolescents (12–18 years old); 3) information about prevalence estimates; 4) dental caries were diagnosed by both primary and permanent definitions (dmft/DMFT/DFS/dfs > 0), according to the WHO criteria; and 5) the study included participants with a validated diagnosis of type 1 diabetes. To assess the quality of articles and to avoid bias, case reports, review articles, case-control studies, letters to the editor, or studies that were conducted on young adults were excluded. However, we included conference abstracts that provided available information about the prevalence of dental caries in children and adolescents with type 1 diabetes. To ensure accuracy and completeness, two co-authors (Y.W. and L.X.) independently reviewed and selected relevant studies, and achieved an inter-rater reliability (Kappa = 0.68). Any differences between the two reviewers were resolved by consensus and a final list of articles was compiled.

### Data extraction and quality assessment

The data were extracted from the list of articles described above. If multiple studies were performed on the same population, the study with the most complete and updated dataset was retained. The extracted information include the name of the first author, year of publication, country, sample size, sex, age, metabolic control of type 1 diabetes, and the prevalence of dental caries. The quality of each study was assessed using the modified Newcastle-Ottawa Quality assessment scale (NOS) [23]. Studies with a NOS ≥ 3 were regarded as high quality (Additional file 3).

### Statistical analysis

All statistical analyses were undertaken using the R software, version 3.5.2. The prevalence of dental caries was extracted from individual studies and combined using the generic inverse variance method of Der Simonian and Laird random-effects.

model. The heterogeneity of effect size among studies was quantified using *I*^2^ [24]. The influence analysis was performed by removing one study at a time to evaluate whether the pool estimates could have been altered by a single study. Subgroup analyses were conducted by mean age, continent and metabolic control of type 1 diabetes. For all comparisons, the pooled prevalence with 95% CI were reported. The Egger’s test and funnel plots were conducted to assess the existence of publication bias. A *p* < 0.05 was considered statistically significant.

## Results

### Literature search and characteristics of the included studies

**Fig. 1:**
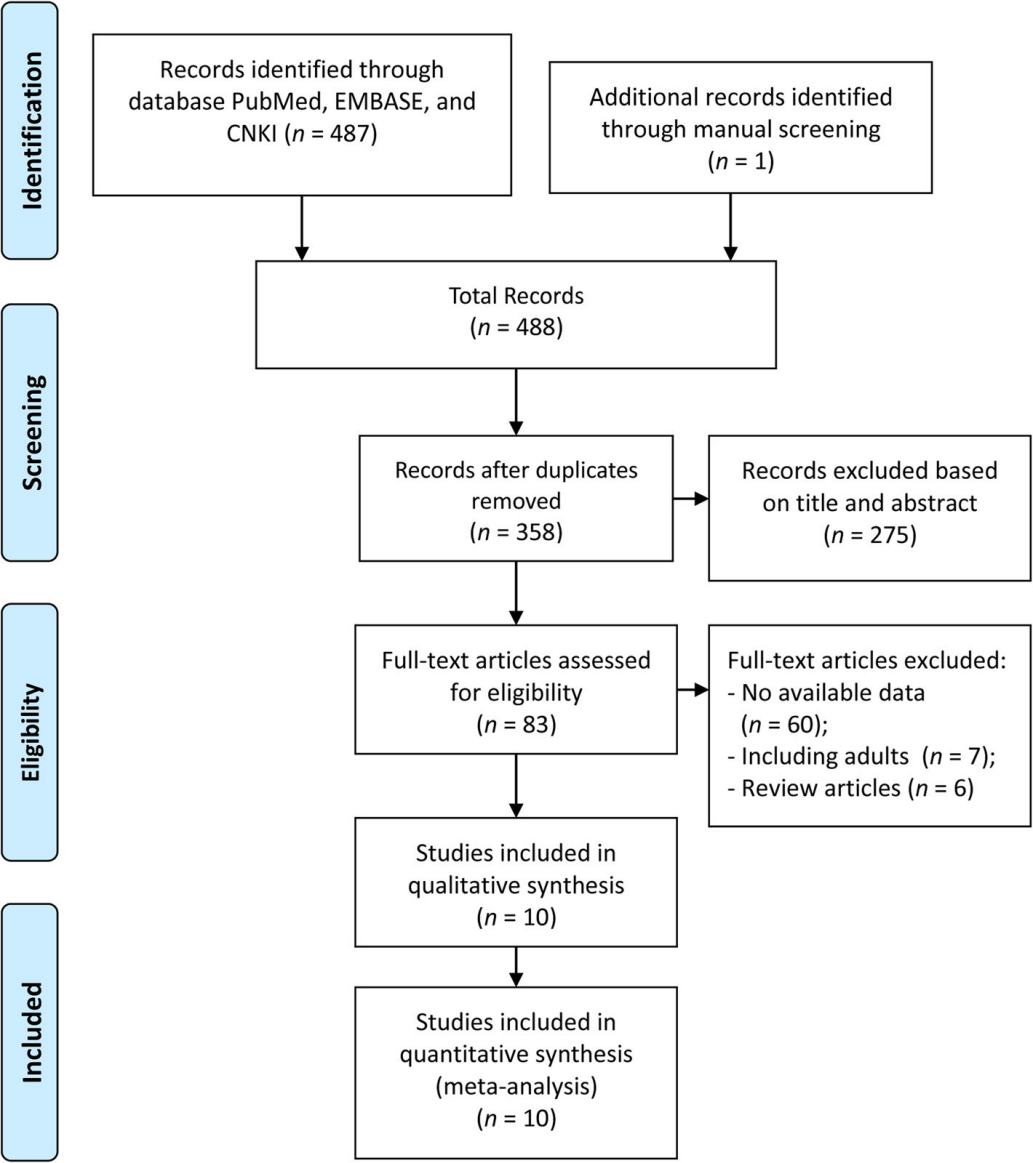
Flow diagram of the literature search

We identified 488 potentially eligible articles, including one article from the reference lists of articles of interest. After the removal of duplicates and screening the titles or abstracts, a total of 83 full-text articles were reviewed, of which 73 articles were excluded. Overall, 10 articles (538 individuals) were included in our final meta-analysis [18–21, 25–30]. A summary of the literature review and study selection process is shown in Fig. 1. Studies were published from 1997 to 2017 with sample sizes ranging from 25 to 87. Among the included articles, four were conducted in Asia, three were performed in Europe, two were conducted in South America and one was conducted in North America. The evaluation of dental caries was conducted through clinical examinations, in accordance with WHO criteria (including DMFT, dmft, DFS, dfs). Information about the metabolic control of type 1 diabetes was provided in three articles.

The methodological quality of studies was based on the modified NOS scale, in which studies could receive up to 5 possible points. One study received 4 points, six studies received 3 points, and three studies received 2 points. The detailed characteristics and quality assessments of those studies are shown in Table 1 and Additional file 4, respectively.

**Table 1:** Characteristics of included studies

Authors, year	Country	Age (years)^a^	Gender (M%)	Total Sample size	Dental caries measurement	Sample size of type 1 diabetes	Sample size of dental caries	Mean (SD) DMFT/dmft	Prevalence of dental caries
Lai et al., 2017	Sweden	12.11 ± 2.77	M/F (48.53%)	204 (136 healthy control)	DMFT	68 GMC (Hb1ac≦7.5): 20 BMC (Hb1ac > 7.5): 48	28 GMC: 6 BMC: 22	NR	41.18% GMC: 30% BMC: 45.83%
Abeuova et al., 2017	Kazakhstan	< 18	NR	60	DMFT	60	47	8.7 (2.2)	70%
Ofilada, 2015	Philippines	10–18	M/F (44%)	25	DMFT	25	18	4.6	72%
Carneiro et al., 2015	Brazil	10.7 ± 2.6	M/F (32.18%)	87	DMFT+dmft	87 GMC (Hb1ac≦8): 11 BMC (Hb1ac > 8): 76	69 GMC: 6 BMC: 61	GMC = 0.8 BMC = 2.4	79.31% GMC: 54.55% BMC: 80.26%
Ofilada et al., 2013	Philippines	1–15	M/F	28	DMFT	28	22	8.2	78.58%
Miranda et al., 2013	Chile	< 15	M/F (68.00%)	25	dmft	25	23	NR	92.00%
Gomez-Diaz et al., 2012	Mexico	11.6 ± 3.2	M/F (52.17%)	69	DMFT	69 GMC (Hb1ac≦7): 11 BMC (Hb1ac > 7): 76	44 GMC:13 BMC: 31	NR	63.77% GMC: 56.52% BMC: 67.39%
Alavi et al., 2006	Iran	11.72 ± 3.36	M/F (44.00%)	50	DMFT	50	18	9.6 (4.6)	36%
Twetman et al., 2002	Sweden	11.2 ± 3.0	M/F (50.00%)	64	DFS + dfs	64	41	NR	64.06%
Karjalainen et al., 1997	Finlan	14.8 ± 1.6	M/F (51.61%)	62	DFS	62	41	NR	66.13%

*GMC/BMC*, good or bad metabolic controlled type 1 diabetes; *NR* not report, *DMFT+dmft* decayed, missing or filled teeth index for permanent and primary teeth, *DFS + dfs* decayed or filled surfaces for permanent and primary teeth

^a^Age was described as mean ± standard deviation or range

### Quantitative synthesis

**Fig. 2:**
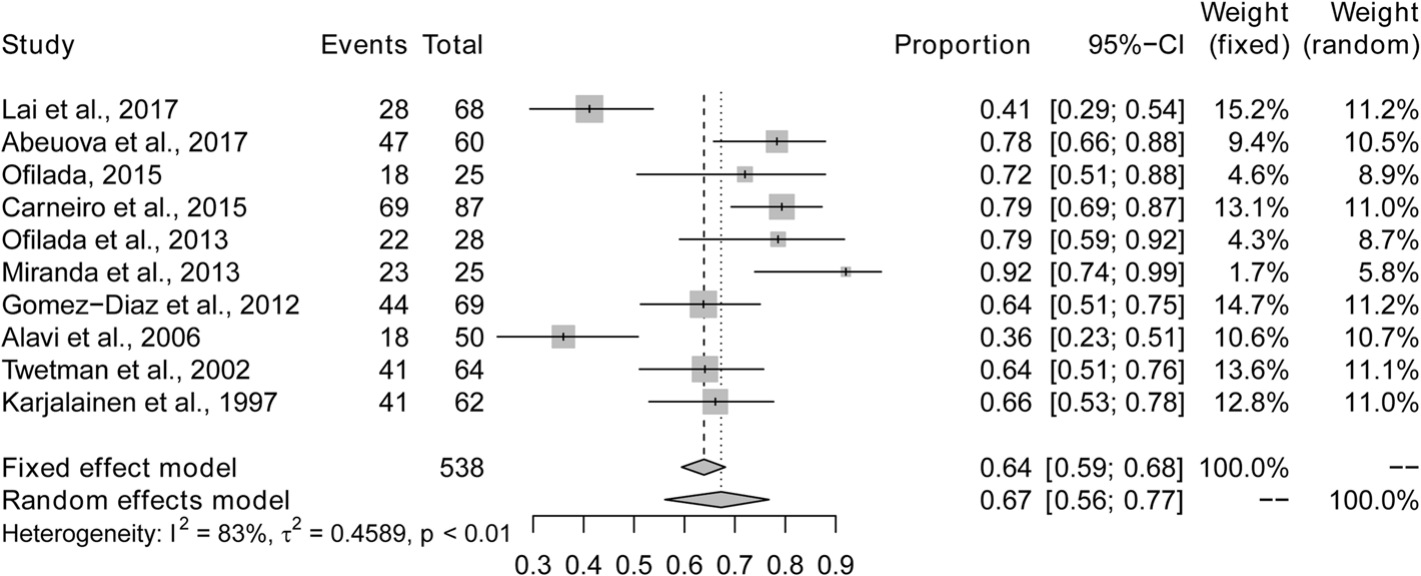
Pooled prevalence of dental caries in children and adolescents with type 1 diabetes

The overall prevalence of dental caries among children and adolescents with type 1 diabetes was 67% (95% CI: 0.56–0.77%; *I*^2^ = 83%) based on the random effects model. The maximum and minimum prevalence of dental caries were observed in Chile (92%) and Iran (36%), respectively (Fig. 2). Five studies provided data of the mean DMFT/dmft. The pooled mean (SD) DMFT/dmft was 5.7 (3.6).

### Subgroup and influence analysis

**Fig. 3:**
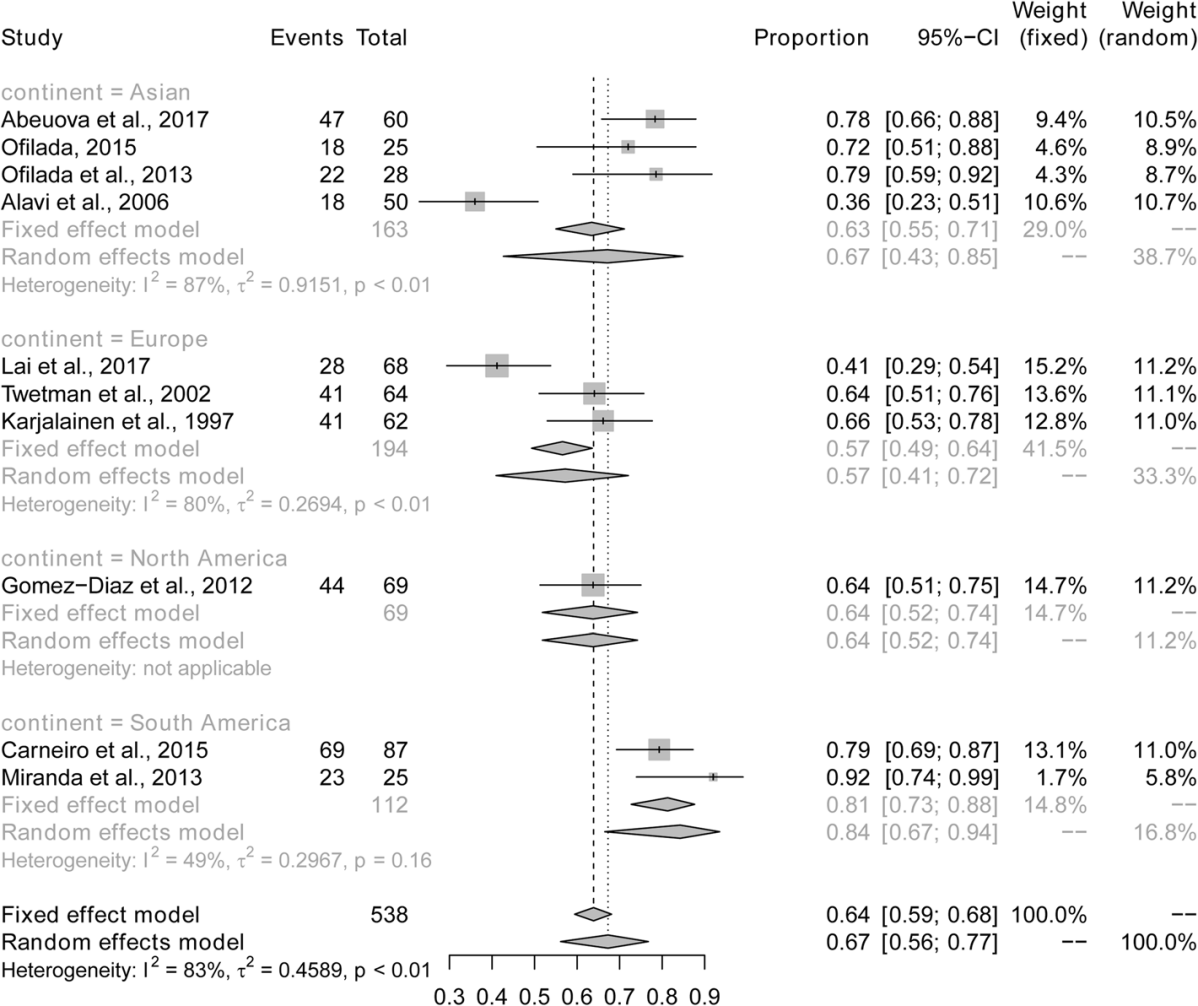
Pooled prevalence of dental caries in children and adolescents with type 1 diabetes according to geographical region

**Fig. 4:**
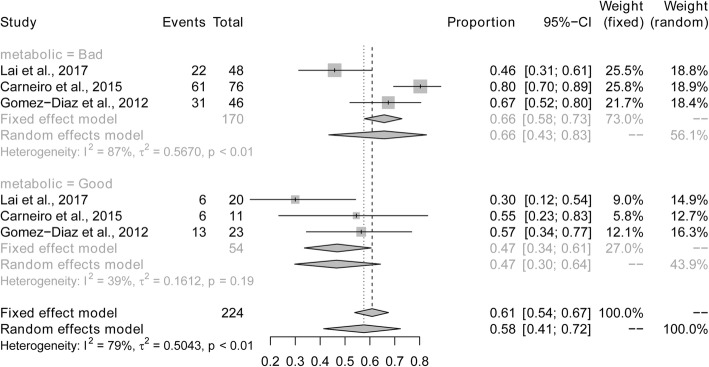
Pooled prevalence of dental caries in children and adolescents with type 1 diabetes according to metabolic control

**Fig. 5:**
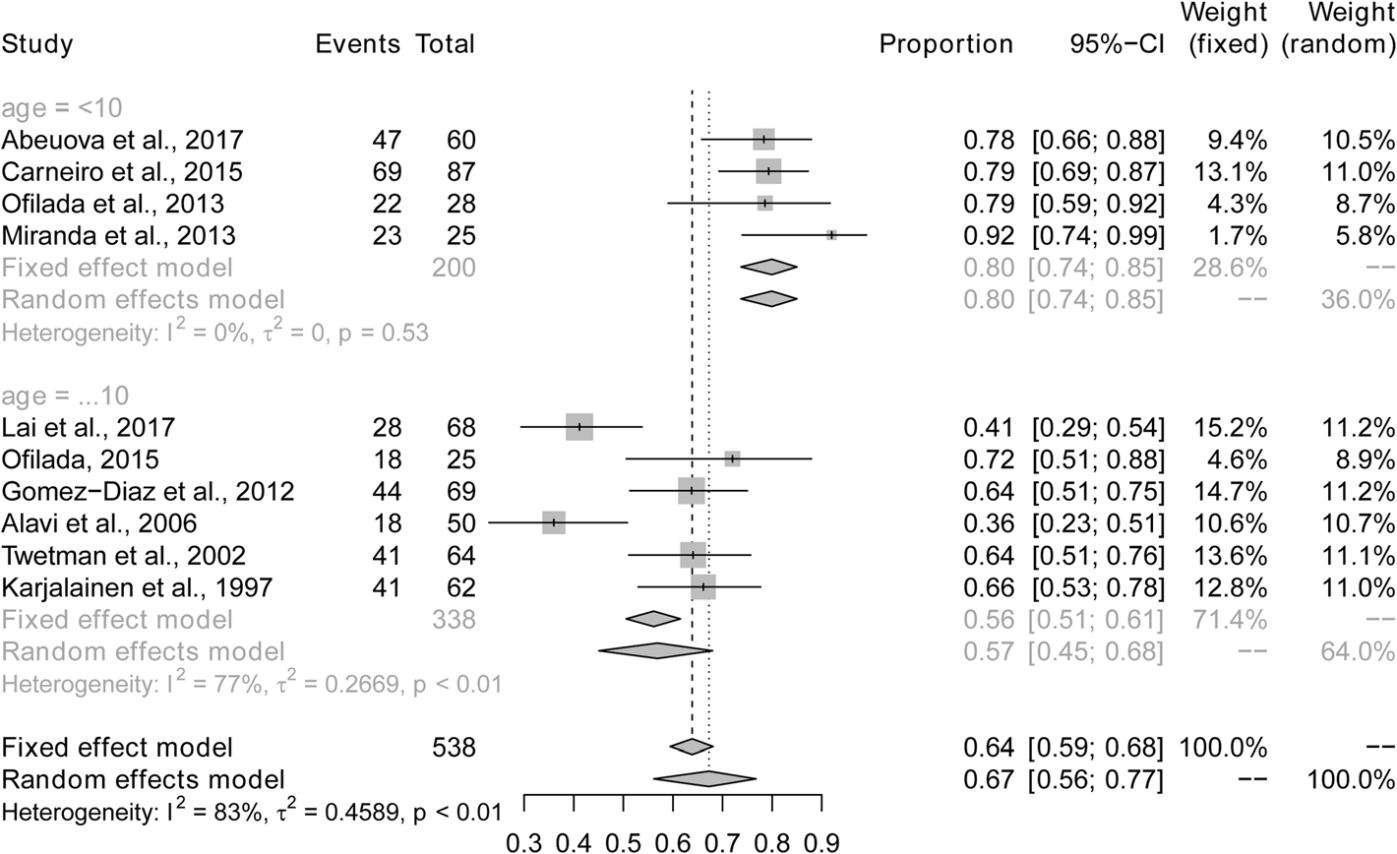
Pooled prevalence of dental caries in children and adolescents with type 1 diabetes according to mean age

**Fig. 6:**
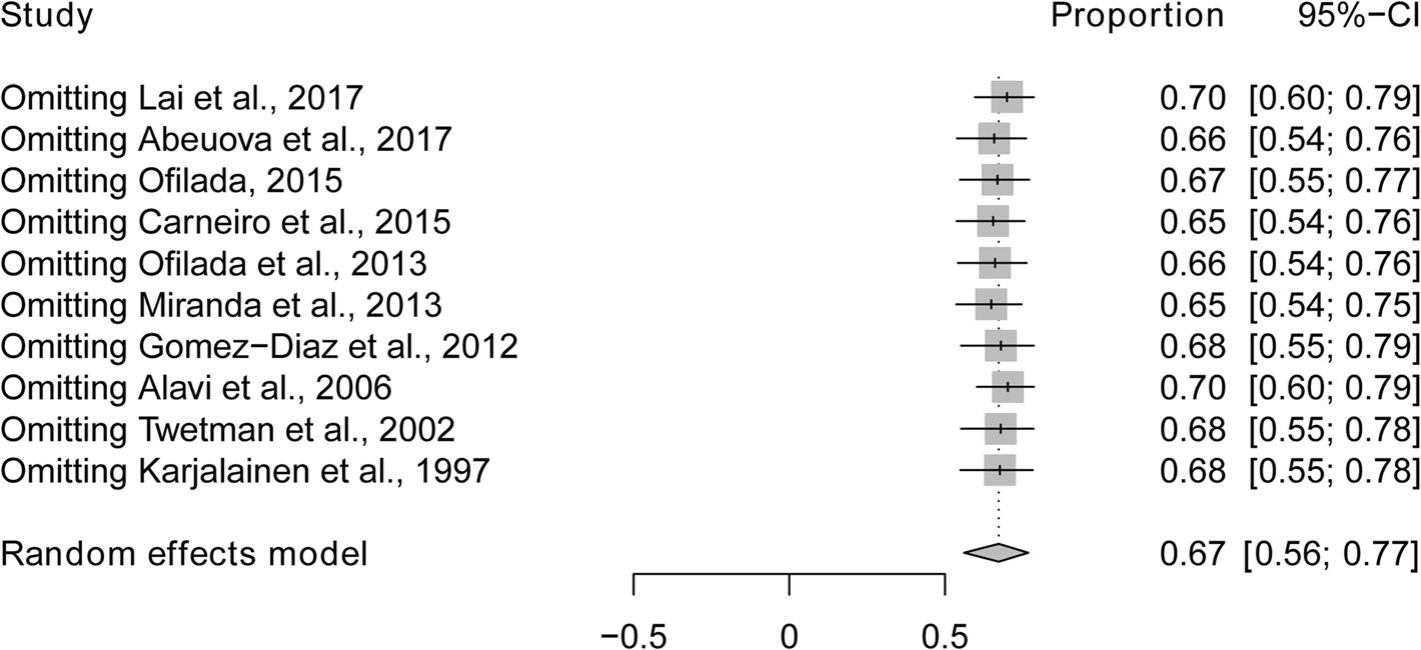
One-study removed sensitivity analysis

Sub-analyses were performed to explore whether heterogeneity might be caused by variations in geographical region or the metabolic control of type 1 diabetes, and whether such differences may also affect the pooled prevalence estimate. We first conducted a sub-analysis among geographical region (Fig. 3). The prevalence of dental caries in South America (84%) was higher than in studies conducted in Asia (67%), North America (64%) and Europe (57%). The next sub-analysis aimed to distinguish between good and poor metabolic control of type 1 diabetes (Fig. 4). The prevalence of dental caries in children and bad adolescents with type 1 diabetes with good metabolic control (47%) was lower than that in children and adolescents with type 1 diabetes with poor metabolic control (66%). Finally, when stratified by mean age (≤10 vs > 10 years old), the prevalence of dental caries was higher in children younger than 10 years of age (80%), compared to children or adolescents older than 10 years of age (56%) (Fig. 5). Sensitivity analyses were performed by omitting one study at a time to judge the robustness of the pooled effect. The pooled prevalence of dental caries ranged from 65 to 70% (Fig. 6). We performed another sensitivity analysis and only retained the studies in which DMFT was used as the diagnostic method for caries, and the pooled prevalence of dental caries was 62%.

### Assessment of publication bias

**Fig. 7:**
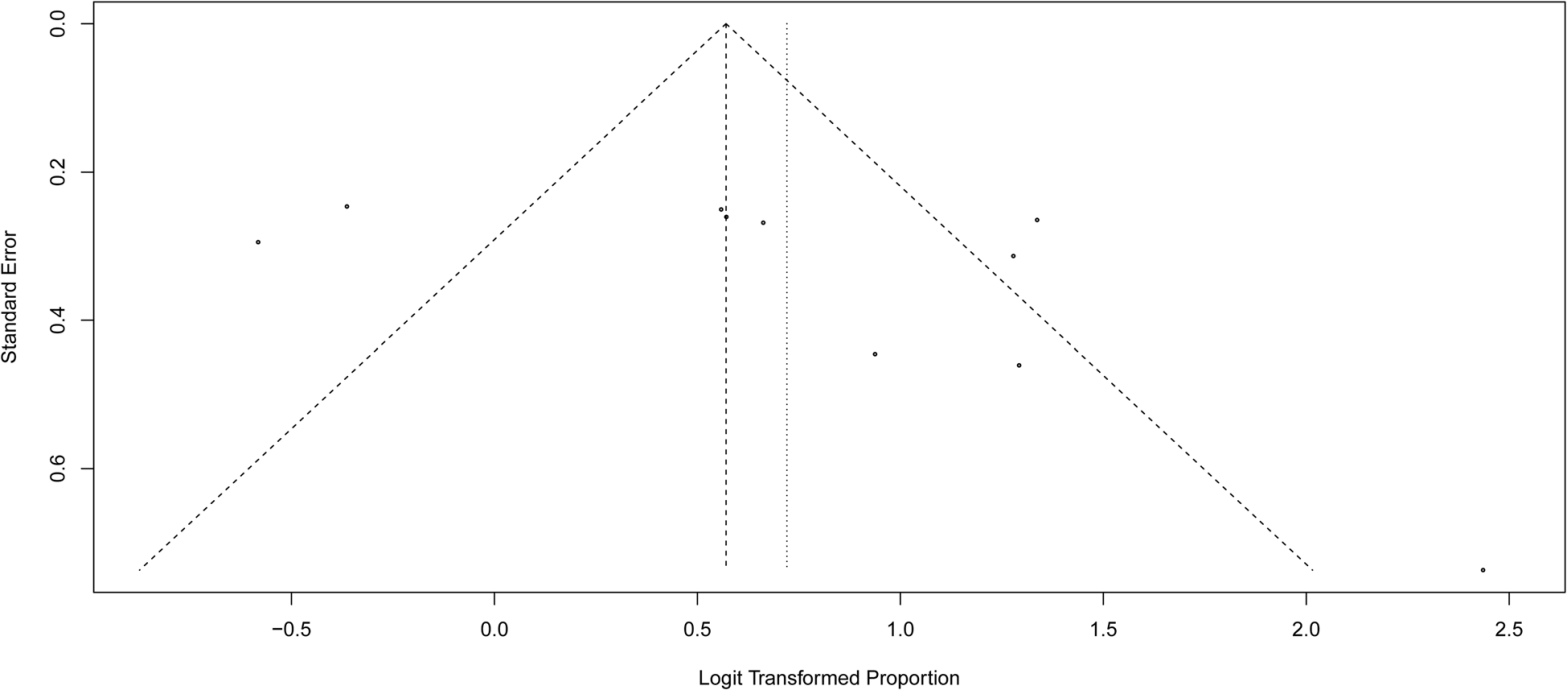
Funnel plot for all studies

We investigated the potential of publication bias using funnel plots and the Egger’s test. Neither methods (funnel plots, Fig. 7; and Egger’s test, *P* = 0.17) showed publication bias in our studies.

## Discussion

Several studies have reported the prevalence of caries among children and adolescents with type1 diabetes; however, studies have shown a high degree of variability in dental caries prevalence among such populations. Thus, this meta-analysis was performed to examine the prevalence of dental caries among children and adolescents with type 1 diabetes. Our results showed that approximately 67% of children and adolescents with type 1 diabetes had dental caries, and the mean DMFT values is 5.7. In a US National Health and Nutrition Examination Survey (NHANES), approximately 37% of children aged 2–8 years and 58% of adolescents had experienced dental caries in primary teeth [31]. Another national survey in Greece showed that the mean dmft/DMFT values were 1.77, 2.05, and 3.19 in 5, 12 and 15-year-old children, respectively [32]. Our study highlighted that the prevalence and severity of dental caries among children and adolescents with type 1 diabetes was higher than that in the general population.

The heterogeneity of dental caries might be explained, in part, by different population characteristics, ethnic backgrounds, diet [33], duration of diabetes, and treatment [34, 35]. In agreement with our results, a study conducted in Mangalore city showed that the prevalence of dental caries was highest in the 5–7 year age group, compared to that of 8–10 and 11–13 year age groups [36]. The higher prevalence of dental caries in younger children might be attributable to the reduction in the number of primary teeth with age due to normal exfoliation. NHANES showed that the prevalence of dental caries among children was 1.5 times higher in Hispanic children, compared to non-Hispanic white and Asian children [31]. Interestingly, a similar result was found in our study, in which the prevalence of dental caries in South American patients was nearly 1.2, 1.3 and 1.5 times higher than that in Asian, North American and European patients, respectively. One research indicated that those patients with a longer duration of type 1 diabetes had higher mean DMFT indexes [34].

As a multifactorial disease, the high prevalence of dental caries in patients with type 1 diabetes, and especially in those with poor metabolic control, might be dependent on the interaction of genetic factors, oral cariogenic bacteria, food intake and oral hygiene. First, insulin deficiency might cause degenerative changes in the salivary glands and lead to a reduced salivary flow and salivary buffer capacity. Additionally, the overall dehydration associated with hyperglycemia might decrease the volume of excreted saliva [37]. Second, Alemzadeh et al., found high levels of *mutans streptococci* and *lactobacilli* among diabetic subjects, particularly in subjects with poor metabolic control [38]. Third, multiple studies showed that a high intake of saturated fat in type 1 diabetic children and adolescents [39–41]. Moreover, consumption of energy dense, low nutrition foods that are characterized by high saturated fat, and low fiber and vegetables have been associated with increased weight gain and obesity, which may lead to dental caries in children [42, 43]. Fourth, although the oral hygiene knowledge and habits of children with type 1 diabetes appear to be superior to healthy individuals [44, 45], poor quality diets might negatively affect oral health through their effects on immune function and glycemic control in children and adolescents with type 1 diabetes [46].

Previous review articles mainly assessed the severity of dental caries in diabetic children and adolescents with type 1 diabetes [1, 47], while our study, investigated the prevalence of dental caries in those populations for the first time. Therefore, this study provided a reference that can be used for clinical consideration, as well as epidemiological and clinical research. Nevertheless, this meta-analysis had several limitations. The heterogeneity was high, although part of which could be explained by geographical region and metabolic control status. Moreover, due to the incomplete data contained in original articles, some factors that greatly influence dental caries, including sex, diabetes mellitus duration and insulin treatment duration, could not be analyzed. Our data captured only 10 studies with small sample sizes, which emphasizes the need for more comprehensive studies worldwide.

Early recognition and intervention can prevent the increased deterioration of dental caries and the potential negative impacts on patients’ quality of life [48]. To decrease the prevalence of dental caries, it is critical for children and adolescents with type 1 diabetes to be screened for early signs of dental caries, and to be examined annually for oral health. Moreover, good metabolic control could help reduce and control the prevalence of dental caries among such populations.

## Conclusion

There is a high global prevalence of dental caries among children and adolescents with type 1 diabetes. Thus, health-care personnel and clinicians should screen and provide preventive treatments during dental clinical routine for diabetic children and adolescents. Further research on the risk of dental caries among children and adolescents with type 1 diabetes is necessary to ensure optimal oral health for such vulnerable patients.

## Additional files

Additional file 1: Search strategy. (PDF 72 kb)

Additional file 2: PRISMA checklist. (DOC 67 kb)

Additional file 3: Modified Newcastle-Ottawa risk of bias scoring guide. (DOCX 12 kb)

Additional file 4: Quality assessment of included studies. (DOCX 15 kb)

## Data Availability

The datasets used and/or analysed during the current study are available from the corresponding author on reasonable request.

## Abbreviations

DMFT: Decayed, Missing, Filled index
NOS: Newcastle-Ottawa Quality assessment scale; PRISMA
PRISMA: Preferred reporting items for systematic review and meta-analysis
WHO: World Health Organization
