# Supplementary material for: Prevalence of dental caries in children and adolescents with type 1 diabetes: a systematic review and meta-analysis
Source: BMC Oral Health. 2019 Sep 14;19:213. doi: 10.1186/s12903-019-0903-5 (PMC6744653; doi:10.1186/s12903-019-0903-5)
Supplement: Supplementary file 1 — Search strategy. (PDF 72 kb) [file 12903_2019_903_MOESM1_ESM.pdf]

**PubMed-140**

| No. | Query | Results | Date |
|---|---|---|---|
| #3 | #1 AND #2 | 140 | 2018-12-28 |
| #2 | ((("Dental Caries"[Mesh]) OR “Dental Decay”) OR “Caries”) OR “tooth decay” | 57,930 | 2018-12-28 |
| #1 | (((((((("Diabetes Mellitus, Type 1"[Mesh]) OR “Type 1 Diabetes Mellitus”) OR “Type 1 Diabetes”) OR “Ketosis-Prone Diabetes Mellitus”) OR “Autoimmune Diabetes”) OR “Juvenile-Onset Diabetes”) OR “Insulin-Dependent Diabetes Mellitus”) OR IDDM) OR “Brittle Diabetes Mellitus” | 91,359 | 2018-12-28 |

**EMBASE-343**

| No. | Query | Results | Date |
|---|---|---|---|
| #7 | #5 AND #6 | 343 | 2018-12-28 |
| #6 | 'dental caries'/exp OR caries OR cariogenesis OR 'dental decay' OR 'tooth decay' OR 'dental fissure' OR 'tooth fissure' OR 'carious teeth' OR 'carious dentine' | 62,140 | 2018-12-28 |
| #5 | #3 OR #4 | 341,821 | 2018-12-28 |
| #4 | 'insulin dependent diabetes mellitus'/exp OR iddm | 110,239 | 2018-12-28 |
| #3 | #1 AND #2 | 341,251 | 2018-12-28 |
| #2 | diabetes | 1,063,113 | 2018-12-28 |
| #1 | brittle OR 'insulin dependent' OR 'type 1' OR juvenile OR 'early onset' OR ketoacidotic | 696,520 | 2018-12-28 |

## China National Knowledge Infrastructure

| No. | Query | Results | Date |
|---|---|---|---|
| #1 | Caries AND “type 1 diabetes” | 4 | 2018-12-28 |
