# Supplementary material for: Prevalence of dental caries in children and adolescents with type 1 diabetes: a systematic review and meta-analysis
Source: BMC Oral Health. 2019 Sep 14;19:213. doi: 10.1186/s12903-019-0903-5 (PMC6744653; doi:10.1186/s12903-019-0903-5)
Supplement: Supplementary file 3 — Modified Newcastle-Ottawa risk of bias scoring guide. (DOCX 12 kb) [file 12903_2019_903_MOESM3_ESM.docx]

**Additional file 3.** Modified Newcastle-Ottawa risk of bias scoring guide.

**1. Representative**

1 point: Sample was original from multiple area.

0 points: Sample was original from single area.

**2. Sample size:**

1 point: Sample size was greater than or equal to 200 participants.

0 points: Sample size was less than 200 participants.

**3. Non-respondents:**

1 point: Comparability between respondent and non-respondent characteristics was established with a satisfactory response rate.

0 points: The comparability between respondents and non-respondents was unsatisfactory, the response rate was unsatisfactory, or there was no description of the response rate or the characteristics of the responders or non-responders.

**4. Ascertainment of dental caries:**

1 point: The evaluation of dental caries was conducted through clinical exam, in accordance with WHO criteria (e.g., DMFT/dmft).

0 points: The evaluation of dental caries was self-reported or there was no description of the assessment method.

**5. Quality of descriptive statistics reporting:**

1 point: The study reported descriptive statistics to describe the population (e.g., age) with proper measures of dispersion (e.g., mean, standard deviation).

0 points: The study did not report descriptive statistics, incompletely reported descriptive statistics, or did not report measures of dispersion.

Legend: Total scores range from 0 to 5. For the total score grouping, studies were judged to be of high quality (≥3 points) or low quality (<3 points).
