# Supplementary material for: Prevalence of dental caries in children and adolescents with type 1 diabetes: a systematic review and meta-analysis
Source: BMC Oral Health. 2019 Sep 14;19:213. doi: 10.1186/s12903-019-0903-5 (PMC6744653; doi:10.1186/s12903-019-0903-5)
Supplement: Supplementary file 4 — Quality assessment of included studies. (DOCX 15 kb) [file 12903_2019_903_MOESM4_ESM.docx]

| **Additional file 4.** Modified Newcastle-Ottawa risk of bias scores. | | | | | | |
| --- | --- | --- | --- | --- | --- | --- |
|  | Representative | Sample size | Non-respondents | Ascertainment of caries | Quality of descriptive statistics | Total score |
| Lai et al, 2017 | **+** | **+** |  | **+** | **+** | 4 |
| Abeuova et al, 2017 | **+** |  |  | **+** |  | 2 |
| Ofilada, 2015 | **+** |  |  | **+** | **+** | 3 |
| Carneiro et al, 2015 | **+** |  |  | **+** |  | 2 |
| Ofilada et al, 2013 | **+** |  |  | **+** | **+** | 3 |
| Miranda et al, 2013 | **+** |  |  | **+** |  | 2 |
| Gomez-Diaz et al, 2012 | **+** |  |  | **+** | **+** | 3 |
| Alavi et al, 2006 | **+** |  |  | **+** | **+** | 3 |
| Twetman et al, 2002 | **+** |  |  | **+** | **+** | 3 |
| Karjalainen et al, 1997 | **+** |  |  | **+** | **+** | 3 |
